# Wellness testing in blood donation: A framework for responsible innovation

**DOI:** 10.1111/trf.70151

**Published:** 2026-03-07

**Authors:** Jeremy W. Jacobs, Sheharyar Raza, Brian D. Adkins, Victoria Costa, David M. Chooljian, Garrett S. Booth

**Affiliations:** ^1^ Department of Pathology, Microbiology, and Immunology Vanderbilt University Nashville Tennessee USA; ^2^ Medical Affairs and Innovation, Canadian Blood Services Toronto Ontario Canada; ^3^ Department of Laboratory Medicine and Pathobiology University of Toronto Toronto Ontario Canada; ^4^ Department of Pathology University of Texas Southwestern Medical Center Dallas Texas USA; ^5^ Department of Pathology NYU Grossman School of Medicine New York New York USA; ^6^ Ethics Consultation Service & Pulmonary & Critical Care Medicine Section, Veterans Affairs Loma Linda Healthcare System Loma Linda California USA; ^7^ Division of Pulmonary and Critical Care Medicine, Department of Medicine Loma Linda University School of Medicine Loma Linda California USA

**Abbreviations**
AABB: Association for the Advancement of Blood and Biotherapies
AMH: anti‐mullerian hormone
HIPAA: Health Insurance Portability and Accountability Act
HIV: human immunodeficiency virus
LLM: large language model
US: United States

## 1 INTRODUCTION

A 45‐year‐old woman donates blood and receives a wellness panel showing elevated liver enzymes. An automated system flags her result as “abnormal” with instructions to “follow‐up with your doctor.” She has no primary care physician. She calls the blood center, who refers her to the third‐party company who actually performed the test. She calls the company, who advises her to “consult a healthcare provider.” Three weeks later, after two emergency department visits and mounting anxiety, repeat testing shows normal values, with the original result attributed to transient elevation from recent exercise. Who is responsible for preventing such a calamity?

This vignette reflects a broader pattern now emerging in transfusion medicine. Over the past decade, direct‐to‐consumer and direct‐access laboratory testing have expanded rapidly, driven by promoters of timely care, increased consumer demand, and for‐profit commercial entities facilitating patient‐initiated ordering.^1^, ^2^, ^3^, ^4^, ^5^, ^6^, ^7^ While these models may improve access for some individuals, broad testing in low‐pretest‐probability populations generates incidental abnormalities and false positives that lead to unnecessary testing, imaging, referrals, and procedures without any clear benefit and possibly introducing unintentional harm.^4^, ^5^, ^8^, ^9^, ^10^, ^11^, ^12^ Additionally, despite enabling patient‐driven decision‐making, the subsequent workload (e.g., result interpretation, follow‐up coordination, patient counseling) falls to clinicians and health systems that did not initiate the testing, would not necessarily have recommended it, and often lack clear follow‐up guidance or reimbursement mechanisms.

Marketing partnerships between blood collection centers and wellness testing companies represent an emerging donor incentive model.^13^ For instance, GoodLabs, which exhibited at the 2025 Association for the Advancement of Blood and Biotherapies (AABB) Annual Meeting, markets wellness panels to blood centers as a donor recruitment and retention mechanism, claiming these “improve show rates, satisfaction, and lifetime value”.^13^ Their “comprehensive” panels assess 75–76 biomarkers covering hematologic, cardiac, endocrine, inflammatory, renal, hepatic, and nutritional parameters, with results delivered via smartphone app. Under this business model, blood centers pay per successful donation, creating a financial incentive structure where wellness testing becomes integral to the recruitment value proposition rather than an incidental benefit. While AABB's exhibitor criteria require professional relevance and subject exhibits to approval, these standards do not evaluate the clinical appropriateness of broad screening panels.^14^ Accordingly, while exhibition does not imply endorsement, presentation in this setting (i.e., the field's flagship meeting) may nonetheless confer perceived legitimacy to such services.

## 2 THE BLOOD SHORTAGE CONTEXT

Blood centers face genuine and pressing recruitment challenges. Ensuring a sufficient blood supply is an essential public health goal, yet blood centers are experiencing sustained declines in new donors and an aging donor base.^15^, ^16^, ^17^, ^18^ Traditional recruitment strategies have shown diminishing returns, and the urgency of maintaining adequate blood inventory drives exploration of novel approaches. Patients need blood, and when standard methods fail to generate sufficient donations, it is understandable that blood centers would consider alternative incentive models—including wellness testing—to attract health‐conscious donors who might not otherwise engage with donation programs.

The appeal of wellness testing as a recruitment tool is straightforward. It offers donors something of tangible value (health information that many in the United States would pay hundreds of dollars to obtain) while potentially attracting individuals interested in preventive health. This is not an unreasonable hypothesis, particularly in the US healthcare system, where many individuals lack consistent primary care access and face significant barriers to routine health monitoring.^19^, ^20^

Notably, “wellness” testing at the time of blood donation is not entirely new. Blood centers have previously explored cholesterol screening as a donor wellness incentive,^21^ yet field experiments evaluating free cholesterol testing found no significant effect on donation rates among either new or lapsed donors, and subsequent large‐scale studies of comprehensive health checks as retention strategies have produced similarly inconsistent results.^22^, ^23^ The intuitive appeal of health testing as an incentive, it appears, does not reliably translate into sustained behavioral change. What has changed is the confluence of scale, immediacy, and communication infrastructure, where large test menus can now be ordered easily, resulted quickly, and delivered directly to a donor's smartphone via automated messaging. In the donation context, this evolution introduces specific risks, including coercive incentives (donate to learn about your health), diagnostic inaccuracy (including perceptions of test characteristics and value to the donor), therapeutic misconception (that testing constitutes medical care), moral hazard (where the testing company and blood center are insulated from downstream harms and thus have weaker incentives to prevent them), and substitution effects driven by inequities in access to care (attracting individuals who lack primary care access and may use donation as a healthcare substitute).^24^, ^25^, ^26^

## 3 WHY BLOOD DONORS FACE UNIQUE VULNERABILITIES

Volunteer blood donation operates in a context that fundamentally differs from typical direct‐to‐consumer testing, creating specific considerations that warrant attention.

First, volunteer blood donation depends on a social contract whereby individuals give freely to help others, and the system protects their safety and privacy in return. This relationship is built on trust. Donors may interpret wellness testing offered during donation as clinically endorsed or medically supervised, not recognizing it as elective consumer screening without formal medical review included in either the pre‐ or post‐testing phases. When abnormal results arrive, donors may expect the blood center to explain their meaning and arrange appropriate follow‐up. Conversely, when they receive “normal” results, donors may feel unduly reassured about their overall health without counseling concerning potential false‐negative results. These expectations create a mismatch between what donors anticipate and what blood centers can deliver. Theoretically, disappointment arising from unmet expectations could diminish the donor‐blood center relationship and reduce future donation activity, paradoxically undermining the very recruitment goal the testing was meant to serve.

Second, blood centers collect blood which is manufactured into products; they do not provide longitudinal medical care and do not have a doctor‐patient relationship with their donors. They generally lack the core elements of primary care infrastructure, such as ongoing patient relationships, integrated medical records, clinical space, referral networks, and liability and staffing models designed for follow‐up and longitudinal management. In addition, standard blood center operations do not yield any insight into whether a donor has a regular source of medical care or not. When wellness testing generates abnormal results requiring interpretation, confirmatory testing, or specialist referral, blood centers will struggle to meet a donor's likely need for guidance and coordination. This challenge is amplified when test menus include assays with limited evidence for screening use or unclear meaning in asymptomatic individuals—tests that many primary care clinicians would not routinely order, and for which even specialists may have limited guidance on how to counsel asymptomatic patients (e.g., leptin; anti‐müllerian hormone [AMH]; fatty acid “OmegaCheck” panels).^27^ Professional society guidance, for example, discourages routine AMH testing in women who are not seeking fertility care, underscoring the risks of deploying specialty‐context biomarkers as general wellness screening.^28^ More broadly, direct‐to‐consumer testing models raise well‐described concerns about the lack of integrated clinical interpretation and the creation of downstream burdens by results that are difficult to contextualize.^4^, ^29^ The fundamental question becomes: who is responsible when a donor receives an abnormal result?

Third, international standards establish voluntary, nonremunerated donation as essential for blood safety.^16^, ^17^, ^30^ While material incentives of varying scale, from tokens of appreciation to substantial prizes such as gift cards, vehicles, and major event experiences, have existed without clear evidence of systematic harm to blood safety,^31^, ^32^ wellness testing occupies a qualitatively different category. Unlike material rewards, wellness testing delivers personalized biological information unique to each donor, data that individuals may actively seek and pay for out of pocket. This personalized dimension may engage a fundamentally different motivational calculus, one that more directly ties the act of donation to individual health gain and may therefore more potently reshape donor motivation in ways that could compromise screening integrity.

These features (i.e., trust‐based relationships, operational limitations, and regulatory constraints) distinguish blood donation from other direct‐to‐consumer testing contexts and suggest that specific safeguards may be warranted.

## 4 CORE CONCERNS

### 4.1 Test‐seeking behavior and screening integrity

When individuals donate primarily to obtain test results rather than to help recipients, they may underreport risk factors during screening interviews, presumably to avoid deferral that would prevent access to desired testing.^33^, ^34^, ^35^, ^36^, ^37^, ^38^, ^39^ This “test‐seeking” behavior is well documented in HIV screening contexts. In one Brazilian study, HIV test‐seeking donors were significantly less likely to disclose risk behaviors compared to repeat voluntary donors.^34^

Wellness testing may create analogous dynamics, but with broader reach. A donor motivated by the prospect of free health panels faces an implicit choice: honestly report factors that might trigger deferral (recent tattoo, medication use, travel history) and forfeit access to testing worth hundreds of dollars, or minimize disclosure and proceed with donation. This pressure operates whether or not donors consciously recognize it, and the transactional framing (“donate to get your results”) may normalize selective disclosure over time, reinforcing the pattern that giving socially desirable answers enables access to desired benefits.

Beyond individual test‐seeking, wellness incentives may reshape the donor pool itself. Motivational crowding theory posits that introducing salient extrinsic incentives can weaken the intrinsic prosocial motives that sustain voluntary behavior, either by reframing the activity in transactional terms or by undermining the donor's self‐perception as an altruistic actor.^40^, ^41^ Evidence for this mechanism in the blood donation context is nuanced. Monetary incentives have shown crowding‐out effects in some settings, while nonmonetary incentives, including conventional health checks, have generally not, with donors tending to perceive them as congruent with the effort of donating rather than as transactional rewards.^42^, ^43^ In charitable giving more broadly, even small tangible rewards can reduce subsequent donations when they draw attention away from altruistic motives toward a cost–benefit mindset.^40^ Contemporary wellness testing, however, may occupy a different position on this spectrum. Unlike a cholesterol result, these panels carry substantial financial value. Whether an incentive of this salience and personal utility operates more like a nonmonetary congruent reward or more like a quasi‐financial benefit remains an open empirical question; the existing literature has not yet directly examined incentives of this type. What can be said is that placing donation as a pathway to personal health gain risks attracting a more test‐motivated donor pool while potentially undermining the altruistic orientation that supports long‐term retention, a risk that warrants prospective study as these programs expand.

If wellness testing programs attract individuals whose primary motivation is obtaining laboratory results, deferral rates may decline not because donor risk profiles have genuinely improved, but because honest disclosure has decreased. Importantly, this dynamic may manifest asymmetrically across the donation pathway, whereby predonation deferrals may fall as at‐risk donors navigate the screening interview, while post‐donation donor deferral and component withdrawal may paradoxically rise as mandatory infectious disease testing detects higher rates of reactive units among collections. The latter scenario carries compounded consequences, as collected units consume processing resources before discard, and in rare window‐period cases may pose residual transmission risk before reactivity is confirmed. This erosion of screening integrity could therefore occur gradually and in ways that aggregate statistics obscure, underscoring the need for prospective monitoring that explicitly tracks donor motivation, self‐reported risk behaviors, and both pre‐ and post‐donation screening outcomes over time.

### 4.2 False positives in healthy populations

Blood donors meet specific health criteria and are generally healthier than age‐matched non‐donors (i.e., the “healthy donor effect”).^44^, ^45^ Most donors lack symptoms or clinical indications for wellness tests being offered. This low pretest probability creates a statistical challenge whereby even highly accurate tests may generate more false positives than true positives when disease prevalence is low. For example, if you screen a population for a condition with 2% prevalence, even with a test with 95% specificity (and high sensitivity), more than two‐thirds of “positive” results are false alarms. Blood centers are already familiar with this concept, as the vast majority of initial reactive infectious disease markers do not confirm.^46^

The donation context amplifies these problems. For blood donors who lack established primary care and pursue donation‐associated testing precisely because they are disengaged from routine healthcare, automated results create additional burdens. Donors who receive abnormal results may face barriers finding appropriate follow‐up. Those who do pursue follow‐up may undergo unnecessary and/or duplicative testing and procedures for findings that prove clinically insignificant, which studies have shown cause substantial anxiety and downstream costs.^47^, ^48^, ^49^ Perversely, the very vendors that conduct the wellness panel screens in partnership with donor centers may be incentivized to induce donors to engage in follow‐up testing, particularly if they offer direct‐to‐consumer testing even outside of the donation context. Donors may share results with blood center staff during subsequent donations, creating informal consultation requests that staff are not equipped to handle. In cases where blood centers do become aware of abnormal results, they may feel obligated to defer donors based on findings that have not been clinically evaluated, reducing supply and donor retention for results that ultimately prove clinically insignificant.

As one physician managing patient‐initiated testing observed, “We all mistake data for knowledge.”^50^ Distinguishing true signal from noise requires clinical judgment, longitudinal data, and follow‐up infrastructure, resources that may not exist in donation‐associated wellness testing programs.

Parallels from employer‐based wellness initiatives are instructive. Workplace wellness programs have historically used screening incentives to drive participation, yet nonselective testing of healthy populations leads to diagnostic cascades and downstream harms with unproven benefits.^51^ The blood donation setting risks recreating these dynamics, but with additional concerns tied to the integrity of the donor screening process and the trust‐based nature of the donor relationship. Even if only 5% of the ~6.8 million Americans who donate blood each year^52^ received optional wellness testing, modest per‐person downstream costs could still translate into millions of dollars in potentially avoidable spending annually, alongside nonfinancial harms such as anxiety, unnecessary procedures, and distraction from higher‐value care.

### 4.3 Accountability gaps and follow‐up

When a donor receives a potentially critical abnormal result, who is legally and ethically responsible for ensuring appropriate follow‐up? If the donor cannot reach the third‐party vendor, does not have a primary care physician, and contacts the blood center for guidance, who provides it? If the donor dismisses the automated message and the finding represents genuine disease, who bears responsibility for the missed diagnosis?

In the United States, hospital laboratories maintain 24/7 critical value policies with documented physician notification and defined escalation pathways. Blood centers have well‐developed infrastructure to respond to urgent donor reactions or product recalls, but these systems do not translate easily to managing abnormal diagnostic test results from wellness panels. Wellness testing platforms may deliver results directly to donors through AI chatbots, automated messaging systems, or asynchronous communications from remote “healthcare providers” the donor has never met. Results may be flagged as “abnormal” or “outside normal range” without clinical context, without knowledge of the donor's baseline values, and without the ability to assess whether findings warrant immediate action versus routine follow‐up versus no action at all.

When donors receive concerning results and contact the blood center for guidance, staff face a challenging position. While many US blood center medical directors have pathology training backgrounds, and thus are well equipped to interpret laboratory values, they are not positioned to provide individualized clinical counseling, arrange follow‐up care, or assume the longitudinal management responsibilities that abnormal results may require. Yet the association between donation and testing may create donor expectations that the blood center can and will provide clinical guidance. This mismatch between donor expectations and blood center capabilities, combined with unclear lines of responsibility between blood centers and third‐party testing vendors, creates potential gaps in accountability when critical or confusing results require follow‐up.

Ultimately, the burden may fall on primary care physicians already managing overwhelming workloads. National reporting illustrates that US physicians are being asked to interpret patient‐initiated laboratory panels and coordinate follow‐up for incidental findings despite limited clinical context and unclear responsibility.^2^ Donation‐associated wellness testing could worsen this problem.

A growing complication is that donors may never seek clinician review at all. Patients are increasingly encouraged to port results into consumer platforms such as smartphone health apps or to upload screenshots into large language model (LLM) tools for “interpretation.”^53^ In this setting, abnormal results can trigger downstream decisions (e.g., additional testing, supplement regimens, self‐medication, care from nonevidence‐based practitioners) without the guardrails of clinical context, confirmatory testing, or duty‐to‐follow‐up frameworks. The result is a widened accountability gap in which neither the blood center nor the vendor controls how results are acted upon once they enter consumer systems, yet donors may reasonably perceive these parties as responsible because the data originated from a donation‐associated partnership.

### 4.4 Data governance and privacy

Blood donation involves informed consent for collection and use of blood products and for testing required to protect recipients and donors. Optional wellness testing is fundamentally different. It is not necessary for donation, generates sensitive information unrelated to transfusion safety, and is often delivered through application‐based platforms with complex data practices.

Wellness testing platforms typically collect demographic data, health history, and longitudinal results valuable for population health research, predictive analytics, or commercial partnerships. Even “de‐identified” data can be re‐identifiable through modern linkage techniques when combined with donation history, geographic location, serial test results, and demographic information. Donors may not realize that language like “improving our services” or “product development” can include creating datasets for licensing to pharmaceutical companies, insurers, or data brokers.

The regulatory landscape offers limited protection. Once health information transfers to third‐party apps or consumer platforms, it may fall outside Health Insurance Portability and Accountability Act (HIPAA) protections. Recent changes in administrative law have increased uncertainty around the scope and influence of agency interpretations on enforcement, leaving consumers with fewer guarantees about how their data will be protected.^54^, ^55^

These privacy concerns carry material consequences. In the United States, while the Affordable Care Act and Genetic Information Nondiscrimination Act limit the use of health status and genetic information in health insurance, these protections generally do not extend to life, disability, or long‐term care insurance, where underwriting commonly considers medical history. Applicants are typically required to answer health questions accurately. Abnormal results generated through optional screening can therefore create future disclosure dilemmas that, if omitted or misstated, may contribute to coverage disputes or policy rescission within contestability periods.^56^ This risk is particularly consequential for young, otherwise healthy donors who never sought testing clinically and may face decades of insurance applications.

The stakes are particularly high in transfusion medicine, where donor trust is strongly linked to willingness to donate blood.^57^ Qualitative research examining donor and non‐donor perspectives on expanded health testing through blood donation found that participants frequently questioned whether such activities fall within the boundaries of what they understood the blood collection agency's role to be, and many were uncertain whether their existing consent adequately covered health information being generated and returned to them.^58^ Acceptability was strongly governed by the perceived beneficiary of the testing, with interventions seen as serving institutional rather than donor interests raising particular concern, and data security was a prominent and recurring apprehension.^58^ Although that work examined genomic rather than biochemical testing, the underlying concerns about role boundaries, consent scope, and who ultimately benefits are highly relevant for wellness panels. Surveys indicate that 81% of US adults are concerned about how companies use the personal data they collect, yet many direct‐to‐consumer testing models rely on lengthy privacy policies and terms of service that obscure rather than clarify data practices.^5^, ^59^, ^60^, ^61^ If donors come to believe their health information was used in ways they did not anticipate or consent to—whether through data sharing with third parties, use in commercial products, or other secondary purposes—the resulting erosion of trust could damage the donor‐blood center relationship and reduce future donation activity. In a system already facing recruitment challenges, privacy breaches or perceived misuse of donor data could worsen the very blood shortage that wellness testing programs aim to address. Blood centers must ensure that wellness testing partnerships do not inadvertently compromise donor privacy or create pathways for secondary data use that donors would not reasonably anticipate when consenting to help save lives.

## 5 A PATH FORWARD

Our goal is not to restrict access to personal health information or undermine patient empowerment to receive appropriate testing. Access to health data can be beneficial, and many individuals, including blood donors, reasonably want more information about their health. In an era where individuals increasingly manage chronic conditions and make healthcare decisions despite limited clinical access, the appeal of actionable health data delivered via smartphone is understandable. Indeed, certain donation‐associated health tests address genuine unmet needs. Ferritin‐guided donation interval programs, now implemented in several countries, offer the clearest example, as rigorous trial evidence from the Netherlands demonstrates that ferritin monitoring in donors, particularly young women, among whom iron deficiency is common, significantly reduces iron deficiency and hemoglobin‐related deferrals while benefiting both donor health and blood center operations.^62^, ^63^ These programs illustrate what responsible donor health testing can look like when the indication is specific and evidence‐based, the result is actionable, and the infrastructure to support follow‐through already exists within the donation context.

The issue is not whether donors should have access to health information, but how that access is enabled and structured, and what infrastructure needs to exist to make wellness testing genuinely beneficial rather than potentially harmful. Empowerment requires interpretable, actionable information delivered within a framework that supports follow‐through. As a field, we should acknowledge both the urgent need to maintain the blood supply and the responsibility to ensure that recruitment innovations do not create new risks for donors or healthcare systems.

## 6 WHAT WOULD RESPONSIBLE IMPLEMENTATION REQUIRE?

If society wants to use wellness testing to support blood donation, several foundational elements would need to be in place. First, any wellness testing program would benefit from clear pathways for result interpretation, follow‐up coordination, and critical value management. This requires either robust partnerships with healthcare systems or dedicated infrastructure within blood centers, resources that would represent significant investment. Without this, the burden falls on donors, emergency departments, and primary care practices.

Test menus would ideally be narrow, evidence‐based, and justifiable rather than comprehensive panels designed for marketing appeal. A thoughtful approach might prioritize assays with clear utility for donation safety and donor health (iron studies, hemoglobin trends) while being cautious about tests with low yield in asymptomatic populations (cardiac biomarkers, hormones, broad metabolic panels). Each included test would benefit from a documented rationale.

Consent for wellness testing should be explicitly separated from donation consent, with clear assurance that donation eligibility is not contingent on accepting testing. This assurance may be reinforced by making the wellness testing explicitly an opt‐in benefit of donation, rather than an assumed or opt‐out benefit. Donors would benefit from transparent information about data collection, storage, access, retention, secondary uses, and third‐party sharing. Current direct‐to‐consumer models often obscure these practices in lengthy terms of service.

AABB and other professional organizations could recognize the need to determine what standards or best practices should apply to donation‐associated wellness testing, acknowledging that current frameworks were not designed with this model in mind. Guidance on test selection, consent processes, critical value policies, medical liability, and outcome monitoring could help blood centers navigate this space more safely.

These elements represent what would be needed to minimize risks, not necessarily what is feasible given current resources and constraints. The gap between what responsible implementation would require and what is currently in place highlights the tension blood centers face: genuine pressure to innovate in recruitment versus limited capacity to manage the downstream complexity that wellness testing introduces.

## 7 CONCLUSION

Donor‐centered innovation is essential, and strategies that support recruitment and retention merit careful evaluation. Blood centers operate under genuine constraints, and declining donor participation creates real pressure to explore new approaches. Wellness testing is being considered precisely because traditional methods have not solved the recruitment challenge, and patients need blood. However, coupling blood donation with optional wellness testing is not a simple solution. Programs that turn the donation experience into a portal for consumer diagnostics may increase short‐term donation, but they risk long‐term harm. The goal is not to discourage innovation, but to ensure that recruitment strategies strengthen rather than compromise the safety and trust upon which blood donation depends. Maintaining an adequate blood supply is critical, but not at the expense of donor welfare or the integrity of the donation system itself.

## FUNDING INFORMATION

No funding was received for this article.

## CONFLICT OF INTEREST STATEMENT

None of the authors report a conflict of interest related to this article. The views expressed in this article are those of the authors and do not necessarily reflect the views of their affiliated organizations.

## Data Availability

Data sharing not applicable to this article as no datasets were generated or analysed during the current study.
